# Fluid-Structure Simulations of a Ruptured Intracranial Aneurysm: Constant versus Patient-Specific Wall Thickness

**DOI:** 10.1155/2016/9854539

**Published:** 2016-09-18

**Authors:** S. Voß, S. Glaßer, T. Hoffmann, O. Beuing, S. Weigand, K. Jachau, B. Preim, D. Thévenin, G. Janiga, P. Berg

**Affiliations:** ^1^Department of Fluid Dynamics and Technical Flows, University of Magdeburg, Magdeburg, Germany; ^2^Department of Simulation and Graphics, University of Magdeburg, Magdeburg, Germany; ^3^Institute of Neuroradiology, University Hospital Magdeburg, Magdeburg, Germany; ^4^Institute of Forensic Medicine, University Hospital Magdeburg, Magdeburg, Germany

## Abstract

Computational Fluid Dynamics is intensively used to deepen the understanding of aneurysm growth and rupture in order to support physicians during therapy planning. However, numerous studies considering only the hemodynamics within the vessel lumen found no satisfactory criteria for rupture risk assessment. To improve available simulation models, the rigid vessel wall assumption has been discarded in this work and patient-specific wall thickness is considered within the simulation. For this purpose, a ruptured intracranial aneurysm was prepared ex vivo, followed by the acquisition of local wall thickness using *μ*CT. The segmented inner and outer vessel surfaces served as solid domain for the fluid-structure interaction (FSI) simulation. To compare wall stress distributions within the aneurysm wall and at the rupture site, FSI computations are repeated in a virtual model using a constant wall thickness approach. Although the wall stresses obtained by the two approaches—when averaged over the complete aneurysm sac—are in very good agreement, strong differences occur in their distribution. Accounting for the real wall thickness distribution, the rupture site exhibits much higher stress values compared to the configuration with constant wall thickness. The study reveals the importance of geometry reconstruction and accurate description of wall thickness in FSI simulations.

## 1. Introduction

Although intracranial aneurysms have been intensively investigated within the last two decades [1], many open questions remain that require further research. Particularly numerical methods are increasingly used since they enable a highly detailed insight into disease processes at no risk for the individual patient. In this regard, Computational Fluid Dynamics (CFD), an established numerical method from classical engineering, was applied to model the blood flow in the human vasculature [2]. Several authors, who investigated patient-specific aneurysm models with regard to intra-aneurysmal flow patterns, identified risk factors for future rupture, but the results are inconsistent. For instance, Xiang et al. [3] associated low wall shear stress (WSS) with aneurysm bleeding, while Cebral et al. [4] detected high WSS within their cohort of ruptured aneurysms. In a subsequent review article by Meng et al. [5], two possible pathways were postulated that assign both low and high WSS a crucial role regarding aneurysm growth and rupture. In addition, Cebral et al. [6] presented a relation between bleb formation and regions of high WSS as well as flow impaction zones.

However, due to patient-individual properties that are unknown (e.g., cerebral flow rates and vital parameters under activity) or due to the requirement of fast computations, all numerical studies are based on several model simplifications. The most severe but commonly used assumption is the treatment of the luminal vessel surface as a rigid, non-flexible wall with infinite resistance. Since three-dimensional segmentations of the diseased dilations are normally gained from contrast-enhanced imaging modalities, only the vessel lumen is represented; no information of the actual wall structure is obtained. However, a study by Frösen et al. [7] has demonstrated the heterogeneity of cerebral vessels, especially when diseases occur.

To extend previous numerical studies by considering mechanical exchanges between blood flow and the surrounding vessel tissue, fluid-structure interaction (FSI) simulations were carried out. Already in 2009 Bazilevs et al. [8] proposed a simple approach to construct vessels with variable wall thicknesses, depending on the radii of inlet and outlet. Cebral et al. [9] used the local WSS distribution of a rigid-wall simulation to estimate the wall thickness, since it induces several pathophysiological processes in the vessel wall. A correlation between the wall thickness as well as its stiffness and the rupture site was presented. The study of Raut et al. [10] focused on FSI simulations of the human aorta. They strongly recommended the use of patient-specific, regionally varying wall thicknesses as well, especially with regard to rupture risk assessment.

Although these studies are important steps towards realistic hemodynamic predictions and FSI simulations in intracranial aneurysms, none of them considered the patient-specific wall thickness. Therefore, the present study is, to the authors' knowledge, the first of its kind that incorporates the measured vessel wall thickness of a ruptured aneurysm into FSI computations. To evaluate the importance of patient-specificity, a simulation assuming constant walls is performed for comparison. The analyses of stress predictions within the complete aneurysm sac as well as at the particular rupture site address the question, whether patient-specific wall thickness is required in related simulations.

## 2. Materials and Methods

### 2.1. Case Description and Preparation

**Figure 1:**
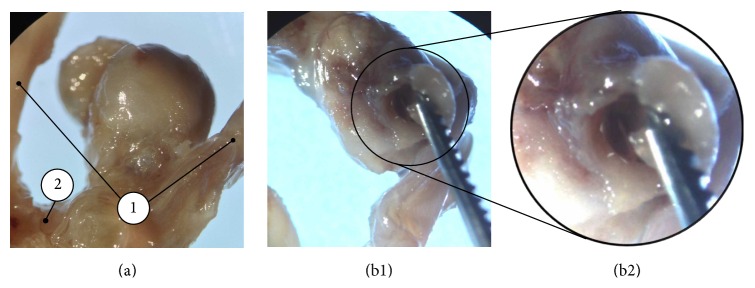
Specimen with Acom aneurysm and adjacent vessels (a). (1) Anterior cerebral arteries. (2) Anterior communicating artery. (b1) Rupture site with magnification (b2).

With approval of the local ethics committee, a complete Circle of Willis (CoW) of a 33-year-old male patient was investigated, which was explanted in the course of a forensic autopsy. Two intracranial aneurysms were found, one at the anterior communicating artery (Acom), the other at the carotid T. Death was caused by subarachnoid hemorrhage due to aneurysm rupture. The Acom aneurysm could be unambiguously identified as the ruptured one, as it was enclosed in a large blood clot and the wall defect was clearly visible (see Figure 1).

To enable the further examination and imaging of the explant, the CoW was put into formaldehyde (4%) for fixation immediately after explantation. Then, the blood clot was carefully removed and the arteries were flushed with formaldehyde. For imaging of the ruptured aneurysm, the anterior cerebral arteries were dissected approximately 10 mm proximal and distal to the anterior communicating artery. After that, plastic tubes were inserted in the anterior cerebral arteries to avoid collapse of their lumen. Plastic was used, because it has a different X-ray density compared to biological tissue and consequently the following postprocessing steps, especially segmentation, are facilitated. The tubes were then stuck into a silicone block in such a way that the specimen had no contact to the silicone surface.

### 2.2. Image Acquisition

For image acquisition, an industrial computed tomography system (Nanotom S 180, GE Measurement & Control, Fairfield, Connecticut, USA) was selected. Despite its low contrast resolution—and thus the impossibility to distinguish different tissue layers of the vessel wall—the device was chosen because of the superior spatial resolution compared to clinical CT and MRI scanners. This allows for the accurate measurement of the wall thickness and visualization of the inner and outer boundary of the specimen. Imaging parameters were as follows: tube voltage of 50 kV, tube current of 150 *μ*A, and reconstructed voxel size of 7.5 × 7.5 × 7.5 *μ*m^3^.

### 2.3. Segmentation

**Figure 2:**
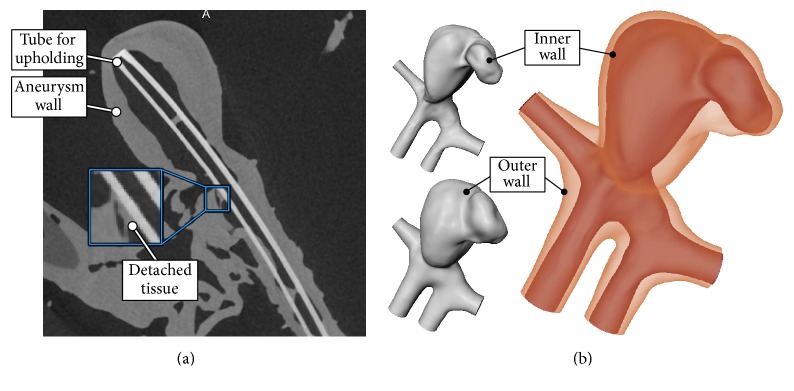
Slice image of the *μ*CT data with the aneurysm wall (a). A detached tissue part of the ex vivo preparation is highlighted (see blue inlay). The resulting surface meshes for the inner vessel wall ((b) top left), the outer vessel wall ((b) bottom left), and the combination of both ((b) right) are illustrated.

Two 3D surface meshes, one of the inner and one of the outer vessel wall, were extracted from the tomographic *μ*CT data. Then, a separate segmentation of both walls was carried out. The workflow is derived from the pipeline for aneurysm surface extraction described in [11]. Initially, a threshold-based segmentation was applied in MeVisLab 2.8 (MeVis Medical Solutions AG, Bremen, Germany) [12]. The initial segmentation was manually corrected with MeVisLab due to the low contrast between vessel wall and vessel lumen of the *μ*CT data as well as small artifacts, for example, detached tissue parts or blood clots, due to the ex vivo preparation. In Figure 2(a), an example is provided, where small detached tissue parts inside the aneurysm are shown.

Next, surface meshes for the inner and outer vessel wall were extracted with Marching Cubes based on the segmentation masks in MeVisLab. Postprocessing of the surface meshes included the manual smoothing of small bumps and artifacts with Sculptris 1.02 (Pixologic, Los Angeles, USA). Furthermore, in- and outlets of the aneurysm were artificially extruded and perpendicularly cut with Blender 2.74 (Blender Foundation, Amsterdam, The Netherlands) to provide sufficiently long enough and straight vessel sections for the subsequent FSI simulation. The resulting 3D surface meshes are depicted in Figure 2(b).

### 2.4. Fluid-Structure Simulations

Since growth and rupture of an intracranial aneurysm are complex problems connecting blood flow and arterial wall behavior, FSI simulations were carried out. Therefore, the segmented aneurysm model was divided into two subdomains consisting of the fluid region and the solid region, respectively. The first was solved numerically using CFD based on a finite volume discretization, while the latter was treated as a structural problem using the finite element method. Both domains were coupled at the interface, the luminal surface. This coupling was implemented as data transfer, exchanging fluid pressure and WSS as well as wall displacement, respectively.

**Figure 3:**
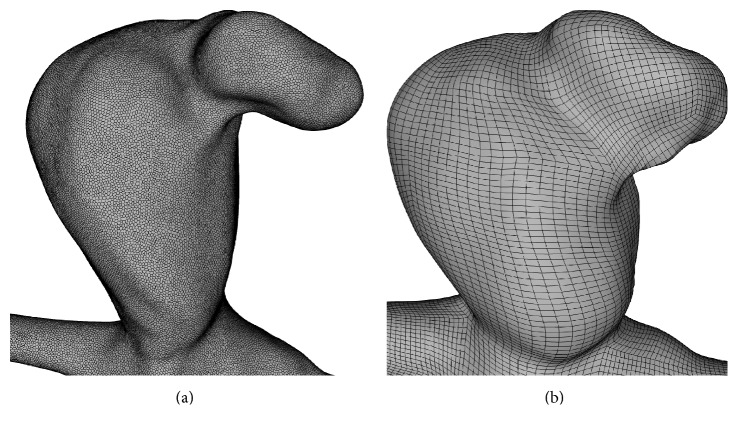
The fluid mesh consists of polyhedral and prism cells (a). Hexahedral finite elements are used for the solid domain (b).

The fluid was modeled as incompressible, non-Newtonian (Carreau-Yasuda model: *η*_0_ = 15.92 mPa s, *η*_*∞*_ = 4 mPa s, *λ* = 0.08268 s, *a* = 2, and *n* = −0.4725, parameters acquired in the local rheology lab) fluid with a density of 1055 kg/m^3^. The inflow conditions were obtained from a healthy volunteer using 7 T PC-MRI [13] and scaled according to the power law of Valen-Sendstad et al. [14]. No-slip conditions for all wall boundaries and zero-pressure outlets were defined. The vessel wall was deformable and coupled the fluid to the solid domain. The latter was assumed to be homogeneous and isotropic using a linearly elastic material model, considering density, Young's modulus, and Poisson's ratio of 1050 kg/m^3^, 1 MPa, and 0.45, respectively [15, 16]. According to Torii et al. [17], this model is reasonable for investigating FSI in intracranial aneurysms. The wall thickness in the constant configuration was set to 0.3 mm according to the mean value for male patients in Costalat et al. [18] and obtained by normal extrusion of the luminal wall. In- and outlets of the solid domain were fixed; all other surfaces were free. The fluid domain was spatially discretized by polyhedral cells and five layers of prism cells at the wall, following the recommendations of Janiga et al. [19]. Regarding the solid domain, a structured mesh and hexahedral elements with quadratic basis function were used. Solvers were STAR-CCM+ 9.04 (CD-adapco, Melville, New York, USA) for the fluid and Abaqus FEA 6.14 (Dassault Systemes Simulia Corp., Providence, Rhode Island, USA) for the solid domain.  Table 1 lists the number of discretization volumes, cells (for the fluid), and elements (for the solid); both are shown in Figure 3.

The time step size for the fluid domain was set to 0.001 s, while the variable time step for the solid domain was limited by the coupling time step of 0.01 s. Two cardiac cycles were simulated, but only the second one was postprocessed to avoid inaccuracies from initialization. The time-dependent FSI simulations were performed on a standard workstation, using four Intel Xeon E3 cores with 3.3 GHz and 32 GB RAM, resulting in calculation times of approximately 30 hours per case.

### 2.5. Qualitative and Quantitative Analysis

Both fluid and solid domain were considered in postprocessing. Streamlines and WSS are presented shortly to provide a qualitative impression of the hemodynamic flow pattern. However, the focus lies on the temporal-averaged stress distribution inside the aneurysm wall, which is initially shown on the inner and outer surface. To carry out a quantitative comparison, wall stress values were exported for two regions of interest: (a) the complete aneurysm sac (approx. 29,000 points) and (b) the rupture site (approx. 6,000 points), which is of particular interest due to its known location. Subsequently, for both regions of interest the spatial-average stress level was calculated and classified into bins of 500 Pa.

## 3. Results

### 3.1. Qualitative Comparison

**Figure 4:**
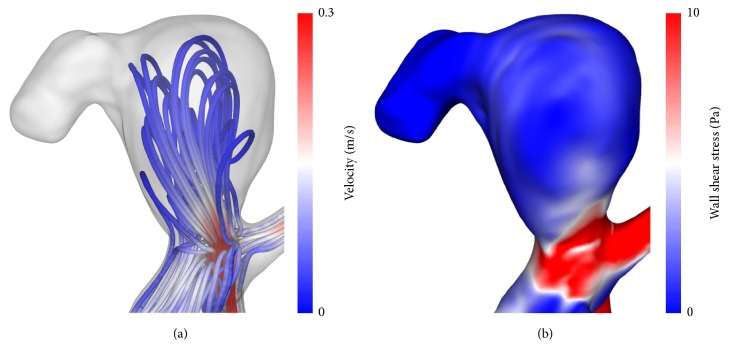
Visualization of the flow pattern (streamlines) and wall shear stresses of the patient-specific configuration at peak-systole.

As presented in Figure 4, the flow velocity inside the aneurysm remains small compared to the parent vessel. This results in low WSS over the entire aneurysm dome. Only in the neck region high values up to 25 Pa are present. Since the time-averaged deformations are below 1 mm, only the patient-specific configuration is shown here.

**Figure 5:**
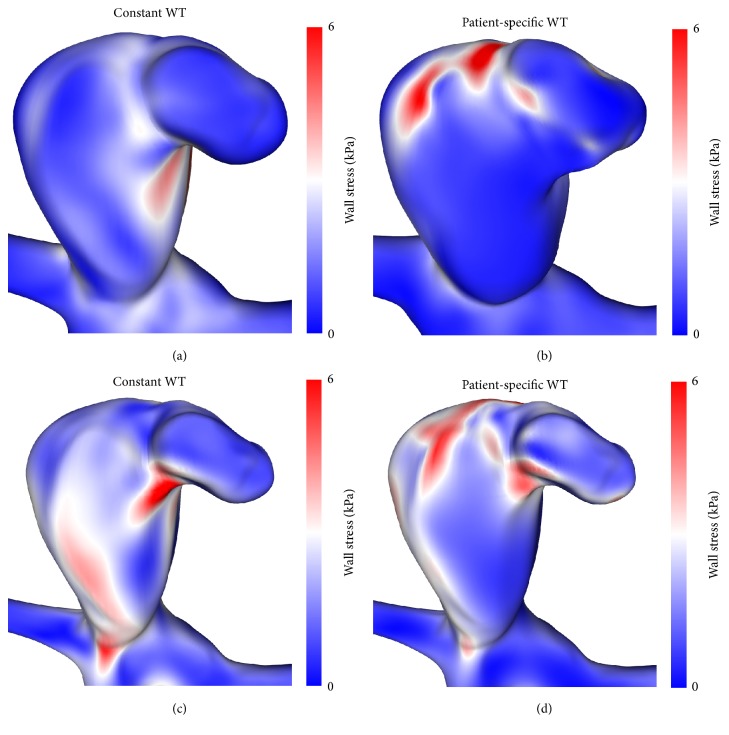
Front view of the effective stress at the outer ((a) and (b)) and inner ((c) and (d)) surface of the constant ((a) and (c)) and the patient-specific ((b) and (d)) wall thickness (WT) configuration, respectively.

**Figure 6:**
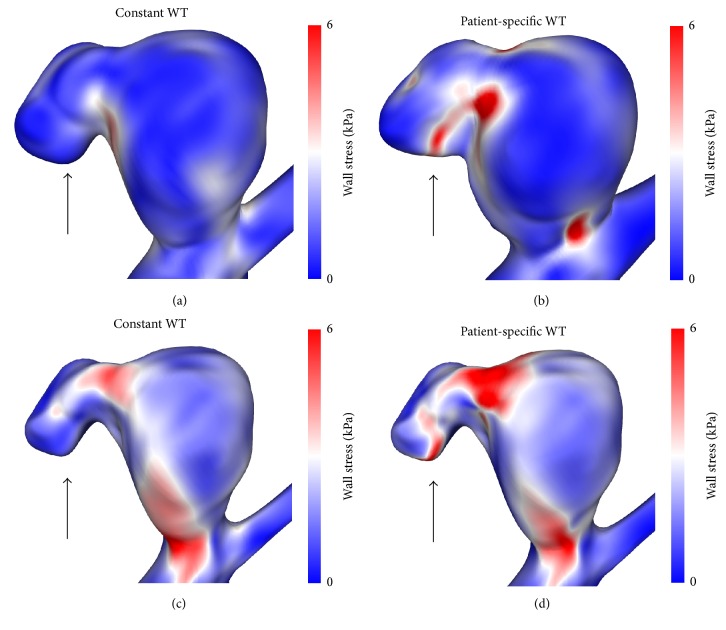
Second perspective of the effective stress at the outer ((a) and (b)) and inner ((c) and (d)) surface of the constant ((a) and (c)) and the patient-specific ((b) and (d)) wall thickness configuration, respectively. Very different stress levels are found at the location of the rupture site (indicated by the black arrow).

The main differences between both configurations concern the effective stress inside the aneurysm wall. Figures 5 and 6 compare the stresses on the outer and inner surface for the constant ((a) and (c)) and patient-specific configuration ((b) and (d)), respectively. Not only do stresses differ in level, but strong local variations are visible as well, leading to different structures. It is particularly interesting to note that the rupture site correlates with spots of high stresses when using the patient-specific configuration (see Figures 6(b) and 6(d)), while nothing particular is observed in this region when a constant wall thickness is used for the computations.

### 3.2. Quantitative Comparison

**Figure 7:**
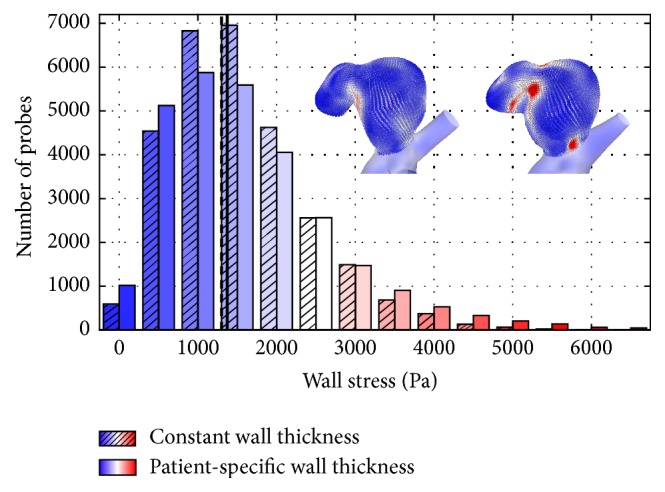
Histogram comparing wall stresses based on approx. 29,000 points in the aneurysm wall. The single bars indicate the number of points in a wall stress range of 500 Pa. As illustrated by the vertical lines, the average stress value obtained with the constant wall thickness configuration (dashed line) nearly matches the level obtained in the patient-specific configuration (solid line).

Figure 7 illustrates the points that are associated with the complete aneurysm sac. Stress values are plotted as histogram plot using bins of 500 Pa. The dashed (constant wall thickness) and solid (patient-specific wall thickness) lines show the high similarity concerning the spatial-averaged stress level of both configurations, in spite of the large local variations. The difference between both approaches is only 3.8% concerning the average, which is not obvious considering only spatial plots.

**Figure 8:**
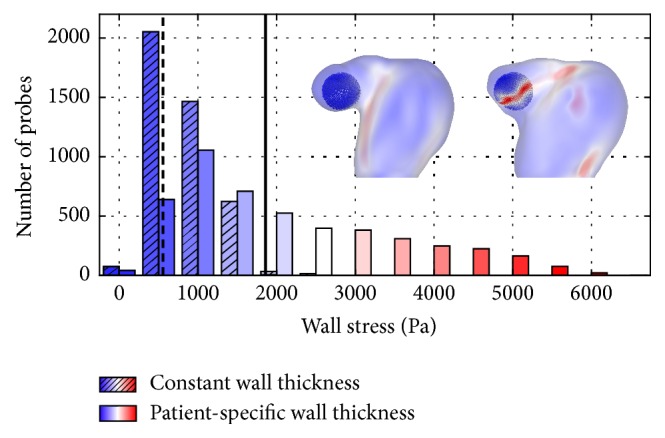
Histogram comparing wall stresses based on approx. 6,000 points around the rupture site. Bars of the constant WT configuration (indicated by the hatching) show a much lower stress level in the rupture zone, compared to the values found with patient-specific wall thickness. The dashed (constant WT) and solid (patient-specific WT) lines depict the mean stress found in the considered region of interest.

To further investigate the aneurysm's rupture site, the quantitative comparison is now concentrated on a smaller region of interest, around the rupture site.  Figure 8 shows the selection of solid grid nodes that are considered for analysis. The histogram plot indicates that values in the constant wall thickness configuration are lower than 3 kPa, while for the patient-specific configuration they reach up to 6.5 kPa in the analyzed area. Likewise, the spatial-averaged stresses (dashed and solid line in Figure 8) reveal a relative difference of 55.2%.

## 4. Discussion

Regarding realistic blood flow predictions in intracranial aneurysms, the reconstructed geometry has an essential impact [3]. In addition, Lee et al. [20] assume that aneurysm morphology is strongly related to aneurysm rupture. Building on these findings, the importance of an appropriate geometry reconstruction for aneurysmal wall mechanics is addressed in the present study. In this regard, a variable vessel wall of a patient-specific intracranial aneurysm and a constant, virtually extruded wall thickness were both considered and compared using FSI simulation. Both investigated configurations are identical with respect to the fluid domain and its conditions. The only difference lies in the wall thickness treatment. This results in almost no differences in the hemodynamic parameters. Therefore, the study focuses on the wall mechanics and particularly on the wall stress, whose distribution varies strongly between both configurations, due to variations in local wall thickness. Consequently, it is suggested that the wall thickness is an important factor for FSI simulations, similar to the lumen morphology in the analysis of flow characteristics.

However, the use of patient-specific wall thickness is limited by the difficulties in acquisition, even ex vivo. This might be one reason for the fact that constant wall thickness is used in almost all similar studies of intracranial aneurysms. Nevertheless, promising models exist, which are related to wall mechanics, but do not take into account the wall thickness itself. Cebral et al. [9] used the value of local WSS to manipulate the local wall thickness and stiffness, respectively. Based on the findings, thin and stiff wall regions in combination with abnormal high WSS correlate with the observed rupture sites. Sanchez et al. [21, 22] used fluid-structure simulations to quantify the volume variations over the cardiac cycle, assuming that material properties have a major impact. Accordingly, large volume variation is caused by weak walls, indicating an increased rupture risk. Still, the application of both approaches for the aneurysm presented in this study might be difficult, since neither the WSS is abnormally high inside the whole aneurysm, nor is it exposed to considerable deformation.

The main focus of the comparison lies in the known rupture site. For the patient-specific wall thickness configuration a good correlation with spots of high stresses is found, contrary to the constant wall thickness configuration. The latter shows a lower averaged stress of 55.2% in the area close to the rupture location. Taking the whole aneurysm into account, high similarity of both approaches in terms of average wall stress is present; the difference is only 3.8%. Accordingly, the choice of wall thickness for the artificial constant configuration is not responsible for the different stress level at the rupture site; it is a direct result of the patient-specific wall thickness. However, it needs to be pointed out that the rupture location does not correlate with the overall highest effective stress value. A reason for that might be the more complex structure of the aneurysm tissue or the surrounding vasculature, which was not considered during the modeling. There might be a general stress level that is dangerous, enabling rupture depending on the wall condition. However, this was not the objective of this study, which only aims at the comparison with constant wall thickness—a common assumption that is often used in FSI computations. Considering this particular case, obvious differences in the local stresses are observed, pointing at limitations associated with the constant wall thickness approach.

Another interesting aspect with respect to the rupture site consists in its location at a daughter aneurysm revealing a bleb-like shape. Cebral et al. [6] investigated the relation between local hemodynamics in particular the WSS and the formation of blebs. According to the authors, blebs mostly occur at or adjacent to aneurysm regions near the flow impaction zone. This assumption is reasonable in this case as well; see Figure 4. In addition, the WSS decreased to a lower level compared to the main aneurysm, as observed by Cebral et al. In the frame of this study, only the final stage of the aneurysm geometry is known and the process of bleb growth remains unclear. However, the stress distribution inside the aneurysm wall may be related to bleb formation. Therefore, patient-specific wall thickness of aneurysm blebs may play an important role to deepen the understanding of this complex process and should be addressed in future studies.

In order to receive numerical predictions with reasonable computational effort, uncertainty and simplifications must be accepted and certainly influence the results. Concerning imaging, vessel position and arrangement as well as fixation differ from the in vivo setting. In addition, the resolution is limited, although *μ*CT offers a good basis for a detailed segmentation process. However, it requires a lot of manual artifact elimination and local smoothing to provide appropriate vessel surfaces. Regarding the inflow condition and wall properties, a representative 7 T PC-MRI measurement and literature values, respectively, were used. It must be kept in mind that the homogenous, isotropic, linearly elastic material model used in this study is far from the real, complex tissue structure found in reality as function of age, activity, location, biological constitution, and so forth. However, Torii et al. [17] pointed out that linearly elastic models may be suitable for corresponding computations.

Future work should take into account a more detailed numerical description of the aneurysm geometry and material. This can be achieved by adding additional information obtained from histology, for example, the distinction between vessel layers and pathologies. The surrounding tissue might play an important role as well and could be considered by specified solid boundary conditions. Finally, a higher number of cases must be included, even if acquiring the real wall thickness is a difficult and time-consuming task.

## 5. Conclusion

The findings of this study highlight the importance of proper geometry reconstruction and accurate description of local wall thickness regarding hemodynamic FSI simulations. The patient-specific wall thickness seems to play an important role for the prediction of stress distributions inside aneurysm walls. While the spatial-averaged wall stresses of the complete aneurysm sac show almost no difference (only 3.8%) compared to those obtained with a constant wall thickness, high differences (55.2%) are observed around the known rupture site. Despite many simplifications, the presented results are a consequent step towards a deeper understanding of aneurysmal wall behavior. Future research is required and should include more cases as well as a more advanced modeling of the wall mechanics.

## Figures and Tables

**Table 1:** Spatial resolution of the FSI computations for the constant and the patient-specific wall thickness configuration.

	Cells in fluid domain	Elements in solid domain
Constant WT	1 206 806	63 072
Patient-specific WT	1 206 806	62 208

## References

[1] Chung B., Cebral J. R. (2015). CFD for evaluation and treatment planning of aneurysms: review of proposed clinical uses and their challenges. *Annals of Biomedical Engineering*.

[2] Berg P., Roloff C., Beuing O. (2015). The computational fluid dynamics rupture challenge 2013—phase II: variability of hemodynamic simulations in two intracranial aneurysms. *Journal of Biomechanical Engineering*.

[3] Xiang J., Natarajan S. K., Tremmel M. (2011). Hemodynamic-morphologic discriminants for intracranial aneurysm rupture. *Stroke*.

[4] Cebral J. R., Mut F., Weir J., Putman C. (2011). Quantitative characterization of the hemodynamic environment in ruptured and unruptured brain aneurysms. *American Journal of Neuroradiology*.

[5] Meng H., Tutino V. M., Xiang J., Siddiqui A. (2014). High WSS or Low WSS? Complex interactions of hemodynamics with intracranial aneurysm initiation, growth, and rupture: toward a unifying hypothesis. *American Journal of Neuroradiology*.

[6] Cebral J. R., Sheridan M., Putman C. M. (2010). Hemodynamics and bleb formation in intracranial aneurysms. *American Journal of Neuroradiology*.

[7] Frösen J., Tulamo R., Paetau A. (2012). Saccular intracranial aneurysm: pathology and mechanisms. *Acta Neuropathologica*.

[8] Bazilevs Y., Hsu M.-C., Benson D. J., Sankaran S., Marsden A. L. (2009). Computational fluid–structure interaction: methods and application to a total cavopulmonary connection. *Computational Mechanics*.

[9] Cebral J. R., Vazquez M., Sforza D. M. (2015). Analysis of hemodynamics and wall mechanics at sites of cerebral aneurysm rupture. *Journal of NeuroInterventional Surgery*.

[10] Raut S. S., Jana A., de Oliveira V., Muluk S. C., Finol E. A. (2013). The importance of patient-specific regionally varying wall thickness in abdominal aortic aneurysm biomechanics. *Journal of Biomechanical Engineering*.

[11] Glaßer S., Berg B., Neugebauer M., Preim B. Reconstruction of 3D surface meshes for blood flow simulations of intracranial aneurysms.

[12] Ritter F., Boskamp T., Homeyer A. (2011). Medical image analysis. *IEEE Pulse*.

[13] Berg P., Stucht D., Janiga G., Beuing O., Speck O., Thévenin D. (2014). Cerebral blood flow in a healthy circle of willis and two intracranial aneurysms: computational fluid dynamics versus four-dimensional phase-contrast magnetic resonance imaging. *Journal of Biomechanical Engineering*.

[14] Valen-Sendstad K., Piccinelli M., KrishnankuttyRema R., Steinman D. A. (2015). Estimation of inlet flow rates for image-based aneurysm CFD models: where and how to begin?. *Annals of Biomedical Engineering*.

[15] Bazilevs Y., Hsu M.-C., Zhang Y. (2010). A fully-coupled fluid-structure interaction simulation of cerebral aneurysms. *Computational Mechanics*.

[16] Valencia A., Ledermann D., Rivera R., Bravo E., Galvez M. (2008). Blood flow dynamics and fluid-structure interaction in patient-specific bifurcating cerebral aneurysms. *International Journal for Numerical Methods in Fluids*.

[17] Torii R., Oshima M., Kobayashi T., Takagi K., Tezduyar T. E. (2008). Fluid–structure interaction modeling of a patient-specific cerebral aneurysm: influence of structural modeling. *Computational Mechanics*.

[18] Costalat V., Sanchez M., Ambard D. (2011). Biomechanical wall properties of human intracranial aneurysms resected following surgical clipping (IRRAs Project). *Journal of Biomechanics*.

[19] Janiga G., Berg P., Beuing O. (2013). Recommendations for accurate numerical blood flow simulations of stented intracranial aneurysms. *Biomedizinische Technik*.

[20] Lee C. J., Zhang Y., Takao H., Murayama Y., Qian Y. (2013). A fluid-structure interaction study using patient-specific ruptured and unruptured aneurysm: the effect of aneurysm morphology, hypertension and elasticity. *Journal of Biomechanics*.

[21] Sanchez M., Ambard D., Costalat V., Mendez S., Jourdan F., Nicoud F. (2013). Biomechanical assessment of the individual risk of rupture of cerebral aneurysms: a proof of concept. *Annals of Biomedical Engineering*.

[22] Sanchez M., Ecker O., Ambard D. (2014). Intracranial aneurysmal pulsatility as a new individual criterion for rupture risk evaluation: biomechanical and numeric approach (IRRAs Project). *American Journal of Neuroradiology*.

